# Genetically predicted associations between circulating cytokines and autoimmune diseases: a bidirectional two-sample Mendelian randomization

**DOI:** 10.3389/fimmu.2024.1404260

**Published:** 2024-05-27

**Authors:** Jie Jie, Yonglu Gong, Songquan Luo, Xing Yang, Kaiyun Guo

**Affiliations:** Changde Hospital, Xiangya School of Medicine, Central South University (The First People’s Hospital of Changde City), Changde, China

**Keywords:** mendelian randomization, cytokine, multiple sclerosis, systemic lupus erythematosus, hashimoto’s thyroiditis

## Abstract

**Objectives:**

Previous studies have indicated a correlation between cytokines and autoimmune diseases. yet the causality remains uncertain. Through Mendelian Randomization (MR) analysis, we aimed to investigate the causal relationships between genetically predicted levels of 91 cytokines and three autoimmune diseases: Multiple Sclerosis (MS), Systemic Lupus Erythematosus (SLE), and Hashimoto’s Thyroiditis (HT).

**Methods:**

A bidirectional two-sample MR approach was utilized to assess the causal relationships between cytokines and MS, SLE, and HT. The datasets included 47,429 MS cases and 68,374 controls, 5,201 SLE cases and 9,066 controls, and 16,191 HT cases with 210,612 controls. Data on 91 cytokines comprised 14,824 participants. Causal analyses primarily employed inverse variance weighted, weighted median, and MR-Egger methods, with sensitivity analyses including heterogeneity and pleiotropy assessment.

**Results:**

Genetically predicted levels of IL-18 (OR = 0.706; 95% C.I. 0.538–0.925), ADA (OR = 0.808; 95% C.I. 0.673–0.970), and SCF (OR = 0.898; 95% C.I. 0.816–0.987) were associated with a decreased risk of MS. IL-4 (OR = 1.384; 95% C.I. 1.081–1.771), IL-7 (OR = 1.401; 95% C.I. 1.010–1.943), IL-10RA (OR = 1.266; 95% C.I. 1.004–1.596), CXCL5 (OR = 1.170; 95% C.I. 1.021–1.341), NTN (OR = 1.225; 95% C.I. 1.004–1.496), FGF23 (OR = 0.644; 95% C.I. 0.460–0.902), and MCP4 (OR = 0.665; 95% C.I. 0.476–0.929) were associated with SLE risk. CDCP1 (OR = 1.127; 95% C.I. 1.008–1.261), IL-33 (OR = 0.852; 95% C.I. 0.727–0.999), and TRAIL (OR = 0.884; 95% C.I. 0.799–0.979) were associated with HT risk. Bidirectional MR results suggest the involvement of CCL19, IL-13, SLAM, ARTN, Eotaxin, IL-22RA1, ADA, and MMP10 in the downstream development of these diseases.

**Conclusions:**

Our findings support causal relationships between certain cytokines and the risks of MS, SLE, and HT, identifying potential biomarkers for diagnosis and prevention. Additionally, several cytokines previously unexplored in these autoimmune disease contexts were discovered, laying new groundwork for the study of disease mechanisms and therapeutic potentials.

## Introduction

Autoimmune diseases (ADs), including multiple sclerosis (MS), systemic lupus erythematosus (SLE), and Hashimoto’s thyroiditis (HT) among others, are a group of conditions characterized by the immune system’s response against the body’s own antigens, leading to self-tissue damage. As complex systemic diseases, ADs may be influenced by factors such as age, gender, and environmental elements (1). It is estimated that MS affects approximately 2.8 million people worldwide, with a prevalence rate of 35.9 per 100,000 individuals (2). SLE exhibits a wide range of prevalence rates—from 13 to 7,713.5 per 100,000—attributable to numerous influencing factors. Regrettably, the incidence of SLE has shown an upward trend over time (3). Meanwhile, prevalence rates for HT are documented between 30 and 150 cases per 100,000 individuals (4). These diseases significantly impair the quality of life for patients and their families, imposing a considerable burden on global health systems and economies. Prior research has highlighted the pivotal role of cytokines in the pathogenesis of ADs, yet detailed understanding of their specific functions and causal relationships remains limited (5). For instance, TNF-α has been identified as a potential biomarker for differentiating SLE patients from controls and as an indicator of disease activity. Elevated levels of IL-16 and IL-10 in lupus nephritis (6), and the immunopathogenesis of SLE, have been suggested to result from the interactions between IL-17 cytokines and effector Th17 cells, T regulatory cells, and B cells (7). Genetic associations with MS risk have been identified for IL-2 receptor subunit alpha (IL2RA), IL7R, CLEC16A, and CD226 (8). Several studies have shown that IFN-γ and TNF-α produced by Th1 lymphocytes recruited in the thyroid tissue induce the release of CXCL10 by thyroid cells, which is responsible for initiating and maintaining the autoimmune process (9, 10). Cytokines are pivotal mediators in the immune system, extensively involved in a range of immune and inflammatory responses. This broad involvement can potentially compromise their specificity as diagnostic biomarkers. However, their integral association with disease pathogenesis remains well-documented (11, 12). Investigating cytokine profiles and their links to specific pathways in various ADs is critically important, as it may significantly enhance our understanding and identification of these complex disorders.

Although previous studies have demonstrated associations between cytokines and ADs, the debate persists on whether cytokines are the cause of ADs, a result of disease progression, or an outcome of medication use. Furthermore, these studies’ conclusions may be affected by unanticipated confounding factors or reverse causality, obscuring the causal relationship.

Mendelian randomization (MR) represents a method to overcome the limitations of traditional epidemiological and observational studies to the greatest extent. It infers the impact of exposure on outcomes by using genetic variants (single nucleotide polymorphisms, SNPs) as instrumental variables in non-experimental data (13). According to Mendel’s laws of inheritance, alleles are randomly assigned during meiosis, unaffected by the environment or lifestyle, thus MR is less susceptible to confounding factors or reverse causality bias, providing more reliable evidence for causal inference (14).

Herein, utilizing recently published genome-wide association study (GWAS) summary data for 91 cytokines (**Supplementary Table S1**) in relation to MS, SLE, and HT, we employ a two-sample MR analysis to (1) explore the potential causal relationships, mechanisms, and therapeutic potentials of cytokines on ADs, and (2) by reversing exposure and outcome, further analyze the association between ADs and cytokines.

## Methods

### Study design

**Figure 1:**
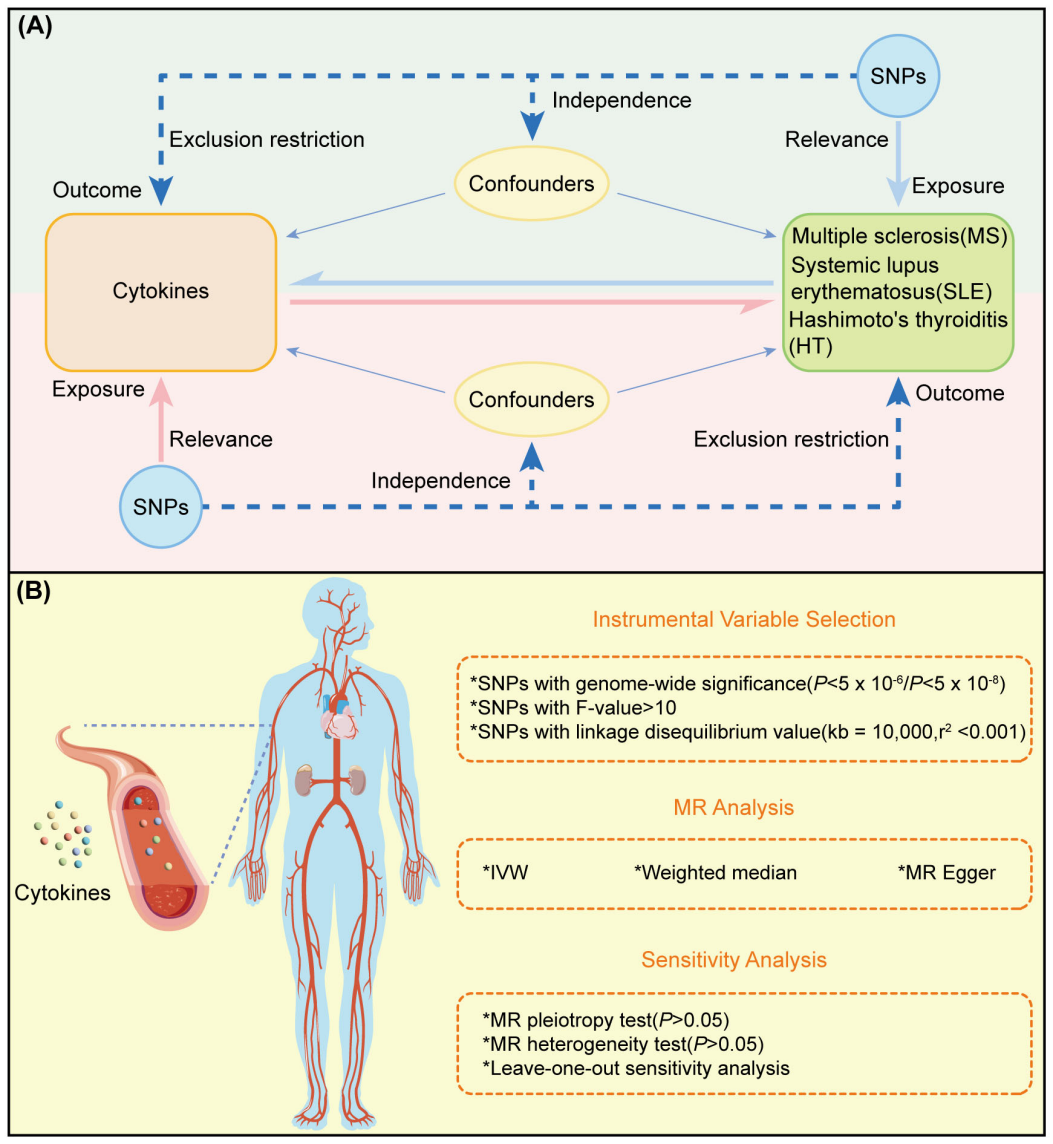
Overview and research content of MR. **(A)** Principle and process of TwoSample MR. **(B)** Specific steps ofTwoSample MR.

The experimental design of this study is illustrated in **Figure 1A**, where the MR analysis requires the chosen genetic variants, serving as instrumental variables (IVs), to satisfy three assumptions (1): IVs are strongly associated with the exposure (Relevance) (2), IVs are not affected by confounding factors (Independence), and (3) IVs influence the outcome solely through their effect on the exposure (Exclusion Restriction). MR analysis and sensitivity testing are employed to evaluate the bidirectional causal relationships between 91 cytokines and three ADs (**Figure 1B**). These cytokines were selected based on their documented relevance to immune responses, as previously reported, and their availability in current databases. Zhao JH et al. performed a genome-wide association study of protein quantitative trait loci (pQTL) on 91 plasma proteins, measured in 14,824 participants using the Olink Target platform (15). The robust methodologies used in this dataset ensure the reliability of the measurements. Furthermore, it includes a wide range of cytokines linked to various immune functions and diseases, representing one of the most comprehensive datasets currently available in public databases. Our study adheres to the requirements of STROBE-MR (16) (**Supplementary Table S2**).

### Data sources

The datasets used in this MR analysis are derived from publicly available GWAS summary data (https://www.ebi.ac.uk/gwas/), negating the need for additional ethical approval. The MS data were sourced from the International Multiple Sclerosis Genetics Consortium (IMSGC), encompassing 47,429 cases and 68,374 controls of European descent. The SLE data originate from research by Bentham J et al., including 5,201 cases and 9,066 controls of European descent (17). HT data were collected from a study by Sakaue S et al., including 15,654 cases of European descent and 37,956 controls, along with 537 cases and 172,656 controls of East Asian descent (18). Finally, gender-stratified GWAS data for the three diseases discussed were obtained from the UK Biobank (UKBB). Unfortunately, comprehensive data encompassing both genders were available only for MS. The datasets for the other diseases were confined to female participants. For detailed information on the UKBB datasets, refer to **Supplementary Table S3**. The exposure and outcome GWAS data were sourced from different consortia to minimize the risk of bias due to sample overlap.

### Instrumental variable selection

Initially, cytokine-related SNPs were selected, with the significance threshold set to *P*< 5 × 10^–8^ to avoid biases from false-positive genetic tools. For exposures with few identified SNPs, the threshold for selecting cytokine genetic tools was raised to *P*< 5 × 10^–6^ (19). Important SNP data were then extracted. SNP clumping (kb = 10,000, r^2^ = 0.001) was performed to remove SNPs in linkage disequilibrium (LD), and, where possible, allele frequency information was used to infer and harmonize the forward strand for palindromic SNPs. Incompatible SNPs were excluded. We estimated the proportion of phenotypic variance explained (R²) and computed the F-statistic within our statistical model. The calculation of R² is defined as 2×MAF×(1-MAF)×β². The F-statistic is determined by the formula $F=\frac{R^{2}}{\left(1-R^{2}\right)}\times \frac{\left(n-k-1\right)}{k}$ , where MAF indicates the minor allele frequency of the SNP, β is the effect size, n is the sample size, and k is the number of IVs utilized. To avoid biases introduced by weak IVs, we excluded SNPs with an F-statistic less than 10, thereby ensuring the reliability and consistency of the retained SNPs (20, 21).

### Statistical Analysis

For AD outcomes represented by binary variables, estimates are presented as odds ratios (OR) and 95% confidence intervals (CIs), reflecting the risk change in ADs per standard deviation (SD) increase in genetically predicted circulating cytokine levels. Bidirectional MR estimates the association between ADs and continuous variable cytokines, with β coefficients and standard errors (SE) indicating the change in SD of circulating cytokines per unit increase in the log-odds of the immune disease (22). The Bonferroni method was used to adjust the evidence threshold for multiple testing (*P*< 0.05) (23), setting it at *P<* 5.5×10^–4^ for forward MR analysis (91 exposures) and *P<* 0.017 for reverse MR analysis (3 exposures).

In our analysis, the primary method used to evaluate causal effects was the Inverse Variance Weighted (IVW) approach, which operates under the assumption that all genetic variants are valid instrumental variables, thereby enabling robust analysis. This method consolidates ratio estimates into a collective causal effect estimate through meta-analysis, boasting the highest statistical power when no pleiotropy is present (24, 25). Additionally, we employed the weighted median and MR-Egger methods to evaluate the reliability and stability of our findings. The weighted median is particularly tolerant of invalid IVs, presuming that reliable estimates are attainable when valid IVs constitute more than half of the total weight (26). This method remains consistent even under potential pleiotropy and has statistical power surpassed only by IVW. Under the Instrument Strength Independent of Direct Effect (InSIDE) assumption, where traditional IV prerequisites are not met but weaker conditions are satisfied, the MR-Egger method also provides an unbiased causal estimate, though it is characterized by lower statistical power and wider confidence intervals (27, 28). Sensitivity analyses, including Cochran’s Q-test and MR-Egger intercept for assessing SNP heterogeneity and horizontal pleiotropy, respectively, were conducted (28). Additionally, the leave-one-out method was utilized to ensure that no single SNP disproportionately influenced the causal inference (29). All statistical analyses were executed using R software (v.4.3.2) and the TwoSampleMR (v.0.5.8) R packages.

We defined robust causal inference as meeting the following criteria: (1) an MR-Egger regression intercept indicating an absence of detectable horizontal pleiotropy (*P* > 0.05); (2) a consistent direction of causal estimates across all methods, with preference given to the statistically strongest IVW estimate in case of discrepancy; (3) leave-one-out results showing no outlier SNPs significantly impacting the causal estimate.

## Results

After removing single nucleotide polymorphisms (SNPs) in linkage disequilibrium and setting the significance threshold at *P*< 5 × 10^–6^, a total of 1,599 cytokine genetic instruments were identified (**Supplementary Table S4**). The range of the F-statistics for the genetic variants was between 18.65 and 4487.22 (**Supplementary Table S5**).

### The impact of circulating cytokines on MS, SLE, and HT

**Figure 2:**
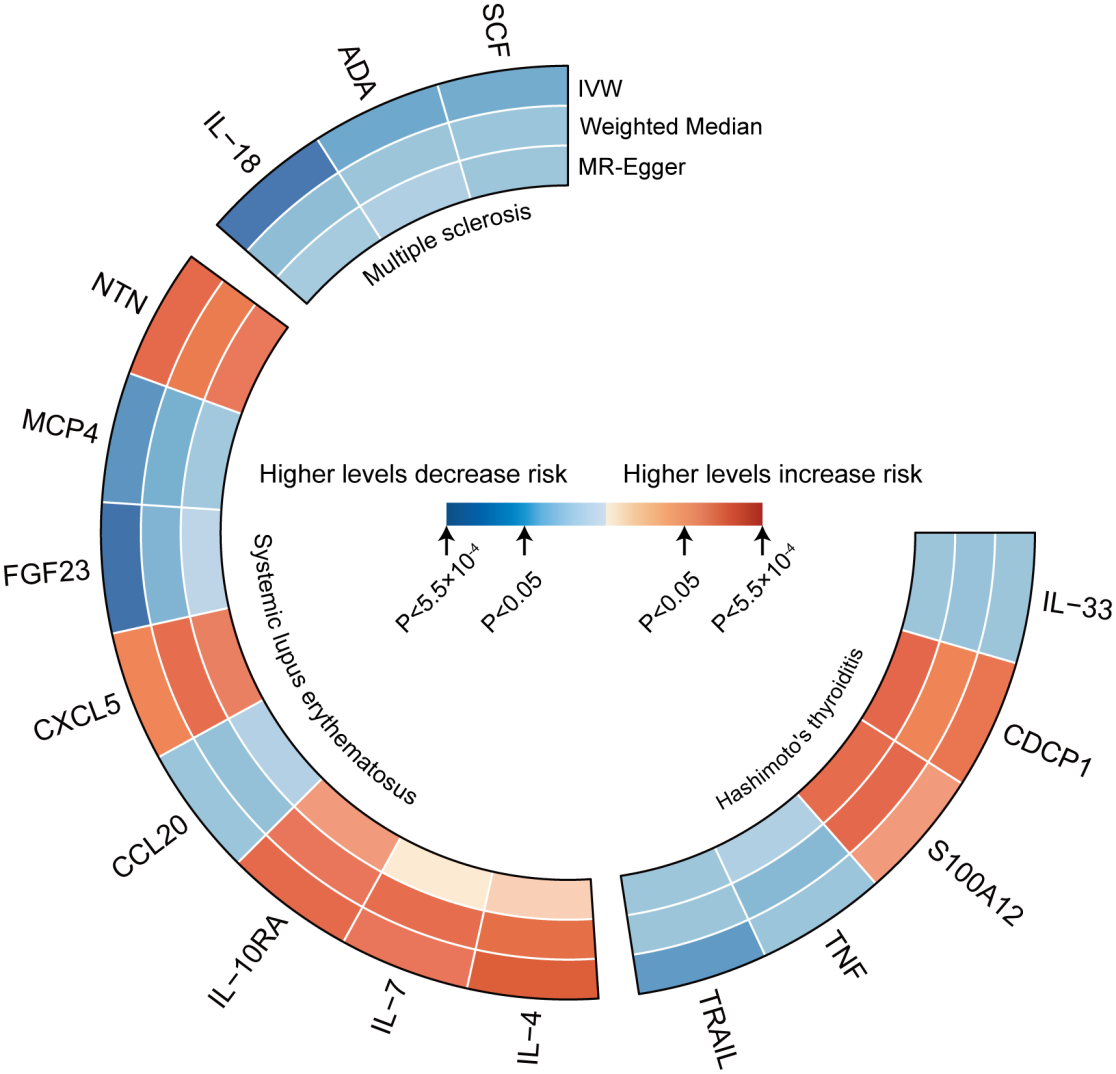
Heatmaps of the different MR analysis methods for the association between cytokines and the risk of three autoimmune diseases.

**Figure 3:**
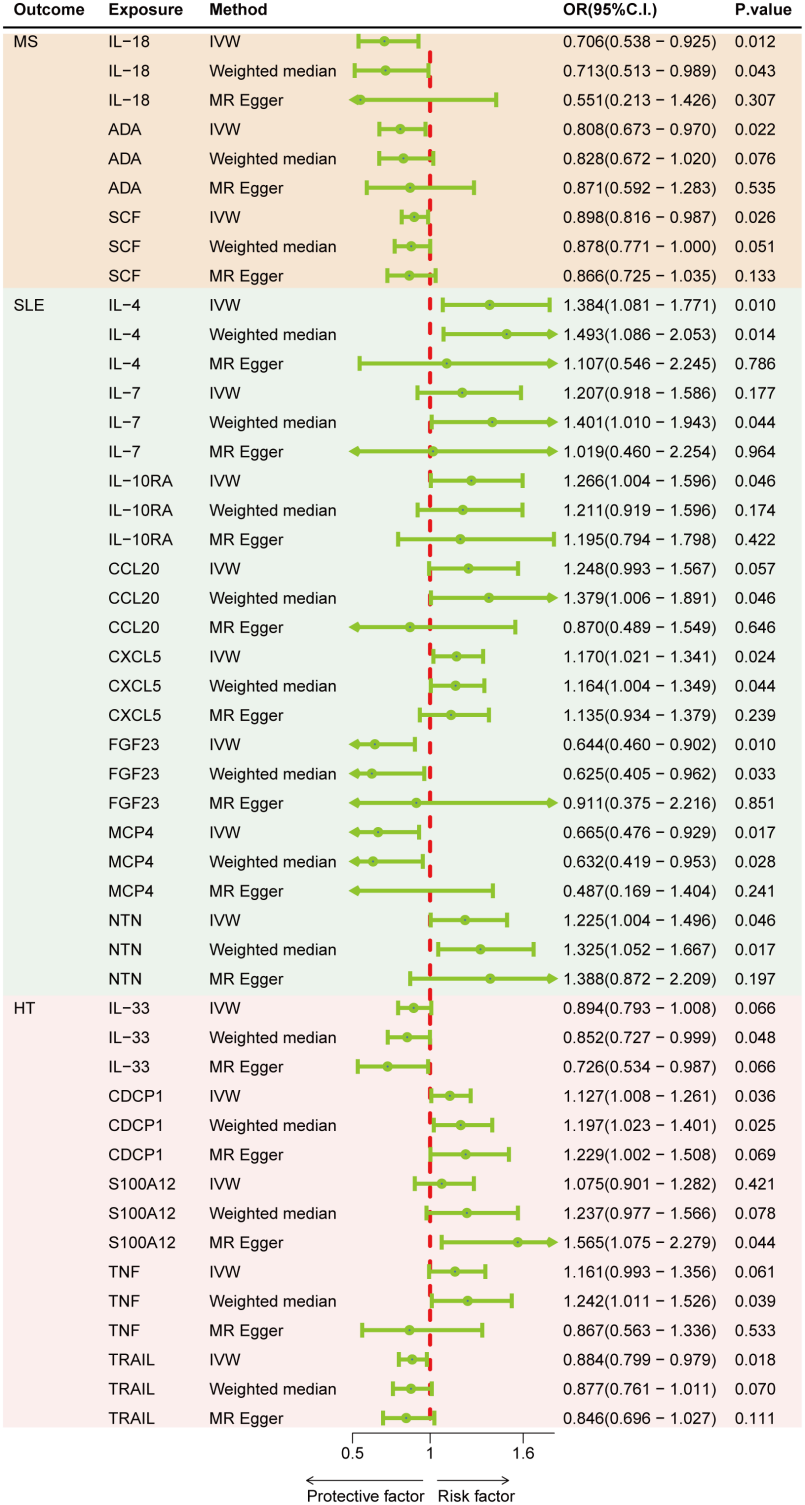
Forest plots of cytokines and the risk of three autoimmune diseases.

**Figure 2** represents a heatmap of associations between circulating cytokines and ADs. According to MR analysis, genetically predicted increases in IL-18 (IVW OR = 0.706; 95% C.I. 0.538–0.925), ADA (IVW OR = 0.808; 95% C.I. 0.673–0.970), and SCF (IVW OR = 0.898; 95% C.I. 0.816–0.987) were associated with a decreased risk of MS per SD increase. For SLE, genetically predicted increases in IL-4 (IVW OR = 1.384; 95% C.I. 1.081–1.771), IL-7 (Weighted median OR = 1.401; 95% C.I. 1.010–1.943), IL-10RA (OR = 1.266; 95% C.I. 1.004–1.596), CXCL5 (IVW OR = 1.170; 95% C.I. 1.021–1.341), and NTN (IVW OR = 1.225; 95% C.I. 1.004–1.496) were associated with an increased risk of SLE per SD increase, while FGF23 (IVW OR = 0.644; 95% C.I. 0.460–0.902) and MCP4 (IVW OR = 0.665; 95% C.I. 0.476–0.929) were associated with a decreased risk of the disease. For HT, a genetically predicted increase in CDCP1 (IVW OR = 1.127; 95% C.I. 1.008–1.261) was associated with an increased risk per SD increase, while increases in IL-33 (Weighted median OR = 0.852; 95% C.I. 0.727–0.999) and TRAIL (IVW OR = 0.884; 95% C.I. 0.799–0.979) were associated with a decreased risk of HT (**Figure 3**). Scatter plots distinctly illustrate the consistency in causal estimates derived from three distinct statistical methodologies (**Supplementary Figures S1**–**S3**), thus excluding CCL20, S100A12, and TNF from the discussion regarding the risk of ADs. Due to the stringent nature of the Bonferroni correction, none of our results remained significant after adjustment, but the consistency in direction suggests suggestive significance. There was no evidence of pleiotropy or heterogeneity between cytokines and ADs (all *P* > 0.05) (**Supplementary Table S6**). The leave-one-out analysis further reinforced the stability of the detection system (**Supplementary Figures S4**–**S6**).

### Bidirectional MR analysis results with MS, SLE, and HT as exposures

**Figure 4:**
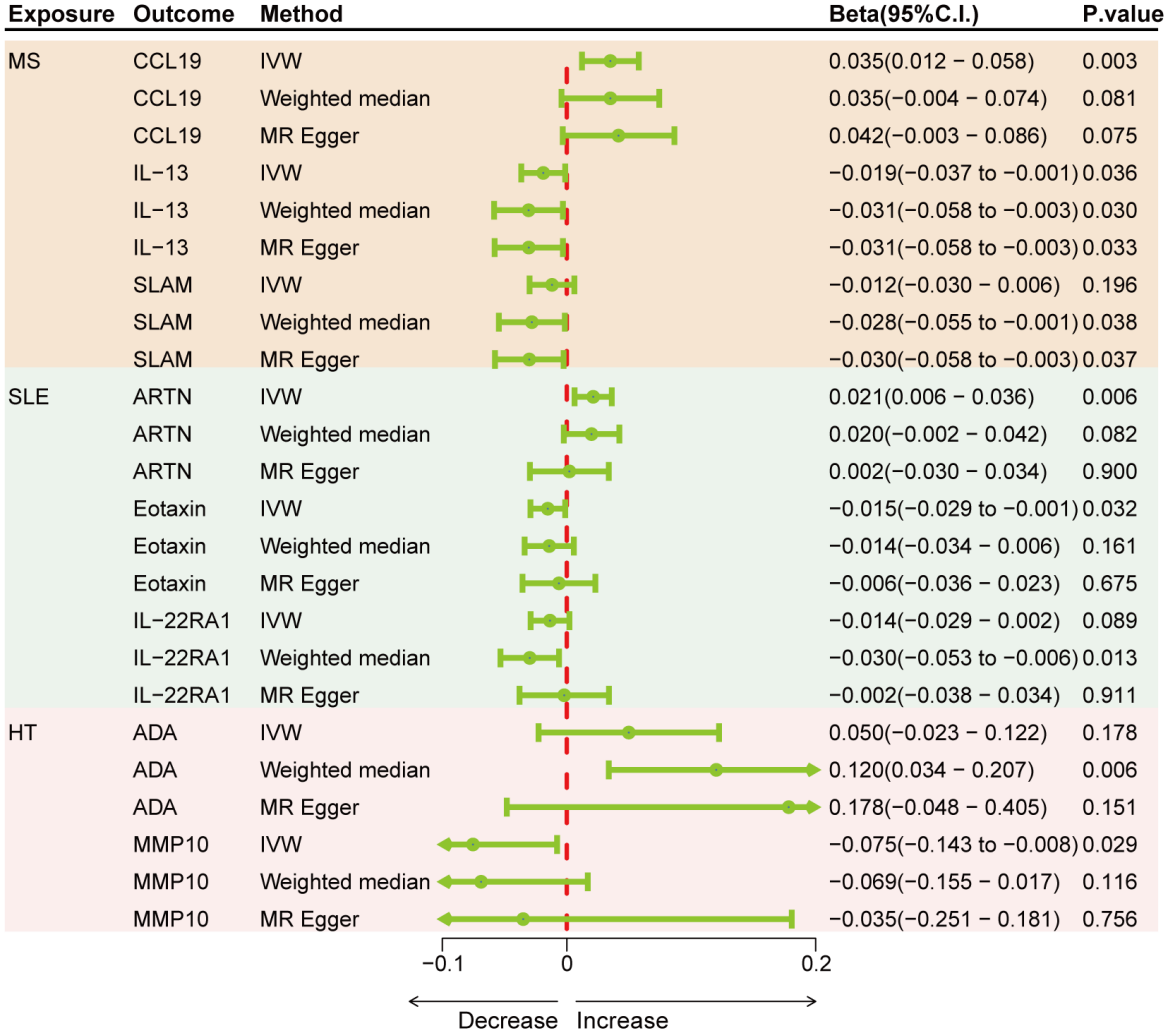
Forest plots showing the effects of three autoimmune diseases on cytokines.

Genetically predicted results indicated that MS is associated with an increase in CCL19 (IVW Beta = 0.035; 95% C.I. 0.012–0.058) and a decrease in IL-13 (IVW Beta = -0.019; 95% C.I. -0.037 to -0.001), SLAM (Weighted median Beta = -0.028; 95% C.I. -0.055 to -0.001). SLE is associated with an increase in ARTN (IVW Beta = 0.021; 95% C.I. 0.006–0.036) and a decrease in Eotaxin (IVW Beta = -0.015; 95% C.I. -0.029 to -0.001), IL-22RA1 (Weighted median Beta = -0.030; 95% C.I. -0.053 to -0.006). HT is linked to an increase in ADA (Weighted median Beta = 0.120; 95% C.I. 0.034–0.207) and a decrease in MMP10 (IVW Beta = -0.075; 95% C.I. -0.143 to -0.008) (**Figure 4**). Although only CCL19, ARTN, IL-22RA1, and ADA remained significant after Bonferroni correction, other results also hold suggestive significance. There was no evidence of pleiotropy or heterogeneity between ADs and cytokines (all *P* > 0.05) (**Supplementary Table S7**). No outliers were detected by leave-one-out method (**Supplementary Figures S7**–**S9**).

### Impact of replicated circulating cytokines on gender-stratified MS, SLE, and HT

Initial MR analyses yielded several positive results. To confirm these findings within gender-specific datasets, further MR analyses were performed. Results revealed a causal relationship for ADA (MR Egger OR = 1.0008; 95% C.I. 1.0001–1.0015) and SCF (MR Egger OR = 0.9983; 95% C.I. 0.9967–0.9999) in females with MS. Similarly, IL-7 (IVW OR = 1.0009; 95% C.I. 1.0002–1.0017) was found to exert a causal effect in females with SLE (**Supplementary Table S8**). Regrettably, no additional positive findings were observed in HT, and SCF was associated with horizontal pleiotropy (**Supplementary Table S9**).

## Discussion

In this study, we utilized the largest available dataset of 91 cytokine GWAS as exposures to analyze their causal relationships with ADs through Two-Sample MR. Our study, conducted within a robust two-sample MR framework, reveals potential causal links between several cytokines and ADs such as MS, SLE, and HT. Specifically, genetic predispositions to elevated levels of IL-18, ADA, and SCF correlate with a decreased risk of MS, suggesting these cytokines have a protective causal role in MS development. For SLE, increased levels of IL-4, IL-7, IL-10RA, CXCL5, and NTN are causally linked to a heightened risk of disease development, whereas higher levels of FGF23 and MCP4 are associated with a reduced risk. In HT, a causal relationship is observed where elevated CDCP1 levels are linked to an increased risk, while increased levels of IL-33 and TRAIL are associated with decreased risk. Additionally, when cytokines were analyzed as outcomes, we found that MS could elevate serum CCL19 levels and decrease IL-13 levels; SLE could raise serum ARTN levels and reduce Eotaxin and IL-22RA1 concentrations; HT was associated with increased ADA levels and decreased MMP10 serum content. From this, we infer that certain biomarkers may be causal in the development of ADs, while some circulating inflammatory factors might be a consequence of the disease progression. Ultimately, our validation of these findings in stratified data revealed that only a limited number of markers were confirmed, and due to several non-artifactual factors, these results are considered somewhat unreliable.

In the context of MR studies, the F-statistic serves as a pivotal measure for evaluating how comprehensively each SNP accounts for variations in the exposure variable. Its adoption as a standard tool for the validation of SNPs as IVs is underpinned by its statistical robustness and its prevalent use in genetic epidemiology research. By opting for strong IVs (F > 10), researchers mitigate the risks associated with Type II errors and biased estimations that are typically linked to the use of weak instruments (21). While alternative indicators such as R² or the T-statistic might be considered, they often fail to offer additional clarity regarding the potential weaknesses of the IVs. The F-statistic is particularly effective in demonstrating the aggregate efficacy of IVs in elucidating the dependent variable, which is crucial in analyses involving multiple variables.

IL-18, which belongs to the IL-1 superfamily, is predominantly synthesized by macrophages and is known to activate the signal transducer and activator of transcription 3 (STAT 3) signaling pathway (30). Studies have documented the crucial role of STAT 3 signaling in oligodendrocytes, particularly in the context of myelin repair (31). This signaling pathway’s involvement is crucially beneficial in addressing the pathophysiology of demyelinating diseases like MS. Despite findings that IL-18 levels are significantly elevated in MS (32), these studies are limited by small sample sizes and unavoidable confounding factors, presenting a contrast to our genetic-level predictive research. This discrepancy underscores the necessity for verification through larger-scale trials. Notably, adenosine deaminase (ADA) levels in the serum of MS patients are significantly higher than those in healthy controls, and there’s a positive correlation between ADA levels and both the clinical activity and disease progression of MS (33). ADA, a crucial enzyme in the adenosine signaling system, plays a vital role in terminating the signaling of extracellular adenosine (ADO), which has immunoregulatory and neuroprotective functions. Research by Vivekanandhan and colleagues found that ADA activity in T cells of MS patients is significantly lower than in controls, during both acute episodes and remission phases (34). This presents a complex picture of ADA’s role, which may vary across different pathological stages or among individuals. Further research is needed to explore its intricate mechanisms. Additionally, we report evidence suggesting a potential causal role of ADA in MS. Stem cell factor (SCF), a dimeric molecule acting through the activation of its receptor, the tyrosine kinase c-Kit (35), is identified in our study as a protective cytokine against MS. SCF signaling plays a pivotal role in regulating microglial functions and facilitating interactions between neurons and microglia (36). Microglia involved in these interactions are known to express glutamate transporter-1, a molecule recognized for its neuroprotective properties (37). Notably, SCF-activated bone marrow-derived cells have been shown to impede the progression of motor neuron diseases. For instance, in Amyotrophic Lateral Sclerosis (ALS) mouse models, transplantation of SCF-activated bone marrow cells has been associated with delayed deterioration of motor functions and prolonged survival (38), although the specific mechanisms remain unclear. Future research in this area could offer breakthroughs for MS treatment.

IL-4, a cytokine associated with type-2 helper T cells (Th2), plays a critical role in stimulating B-cell maturation. As B cells differentiate, they can potentially enhance autoimmune responses through increased production of autoantibodies. In the case of SLE, such mechanisms may be over-activated, resulting in disturbed immune regulation and the promotion of autoimmune responses. This over-activation highlights the complex dynamics within the immune system that contribute to the pathology of SLE (39). Increased levels of IL-4 are found in patients with SLE, and notably, serum concentrations of IL-4 and IL-7 are significantly elevated in children, potentially triggering the inflammatory processes associated with SLE (40–42). Elevated levels of IL-7 may contribute to autoimmune responses in SLE by enhancing T-cell survival and proliferation (43). Similarly, IL-10 levels are higher in SLE patients, highlighting an important link with IL-10RA (44, 45). Our findings align with these observations, although the evidence for IL-7 and IL-10RA is relatively weak. Monocyte chemotactic protein 4 (MCP4), part of the chemokine family. In a cross-sectional analysis, serum MCP4 levels were quantified in patients with systemic sclerosis, dermatomyositis, SLE, and healthy controls. Contrary to expectations, there was no significant decrease in MCP4 levels in SLE patients when compared to the control group (46). While MCP4 is recognized for its antimicrobial activity, which may contribute to the body’s defensive mechanisms, its specific functions and underlying mechanisms in the context of ADs are still not well understood (47). CXCL5, an inducible chemokine, may be involved in various inflammatory diseases (48). Nucleosomes, significant autoantigens implicated in the induction of SLE, lead to a marked upregulation of CXCL5 expression in mesangial cells from the kidneys of nucleosome-stimulated mice (49). In SLE patients, during the immune response phase, CXCL5 plays a pivotal role by recruiting and activating neutrophils and T/B immune cells. This function is critical in the pathophysiological progression of the disease (50). Neurturin (NTN) and fibroblast growth factor-23 (FGF23) have not been reported in SLE-related studies. Future research should aim to identify more genetic variants through larger GWAS and delve into the molecular and pathological mechanisms of these candidate factors to clarify these findings.

Previous studies have identified a novel association between CUB domain-containing protein 1 (CDCP1) and autoimmune endocrine diseases, with CDCP1 levels significantly elevated in patients with HT (51), providing some support for our findings. CDCP1 is recognized as a critical ligand for CD6, a key modulator of T-cell activation and a recognized risk gene for ADs. The interaction between CD6 and CDCP1 is vital for the modulation of T-cell responses in ADs. Additionally, soluble CDCP1 acts as a chemoattractant for T-cells, influencing their migration to inflammatory regions, which is essential for the development and maintenance of inflammatory responses in ADs (52). Celik and colleagues reported increased levels of IL-33, a member of the IL-1 family, in Graves’ disease, suggesting it as a biomarker associated with Th2-related autoimmunity (53). However, this study involved only 25 HT patients and 25 controls, employing enzyme-linked immunosorbent assay (ELISA) for its experimental method. Therefore, the precise role of IL-33 in HT requires further in-depth research. Additionally, only one study has documented the role of tumor necrosis factor-related apoptosis-inducing ligand (TRAIL) in thyroid diseases. Although TRAIL is considered a protective factor in the treatment of thyroid cancer (54), its potential in HT therapy remains an unexplored area, indicating a significant gap in the current understanding and treatment of HT that warrants further investigation.

In reverse MR analyses, we reported that MS leads to increased levels of the chemokine (C-C motif) ligand 19 (CCL19), although the relationship between CCL19 and MS is not entirely clear. Elevated CCL19 levels in the cerebrospinal fluid (CSF) of MS patients are associated with the pathology and disease activity of MS (55), necessitating further investigation to clarify this relationship. IL-13, as an immunoregulatory cytokine from the Th2 family, is endowed with anti-inflammatory properties, especially relevant in autoimmune and allergic environments. Yet, its precise influence on the onset and progression of MS remains elusive, being either beneficial or detrimental (56). Our findings lend genetic support to the hypothesis that MS may lower plasma concentrations of IL-13. Furthermore, in female patients, there is a positive correlation between ARTN and the SLE risk factor Integrin alpha M (ITGAM). Notably, ARTN acts analogously to monocyte dipeptidyl peptidase IV (CD26), present in T/B/NK cells, with its inhibitors emerging as potential therapeutic agents for various autoimmune conditions (57), which lends indirect support to our observations. Additionally, chemokines, a category of chemoattractant cytokines, contribute significantly to the pathogenesis of SLE and its complications, notably lupus nephritis (58). The research by Novikov A, et al., indicates a notably lower expression of Eotaxin in the SLE group compared to those with rheumatoid arthritis (59), highlighting the unique inflammatory profiles in these conditions. IL-22RA1, an essential component of the IL-22 receptor complex, has been observed to decrease in serum and plasma levels as reported by Cheng and Pan (60, 61). The precise mechanisms through which SLE mediates these reductions are not fully understood, but may relate to a restriction in the expression of the heterodimeric receptor complex that includes IL-22RA1, impacting the signaling process (62). In the context of HT, an increase in ADA levels has been noted. Given the established associations of ADA with cancerous processes and binding interactions (63, 64), the ADA elevation observed in HT may be implicated in similar pathways, suggesting a potential avenue for further research into the pathophysiological implications of ADA in autoimmune thyroid disorders.

Clinically, some progress has been made in the treatment of ADs, yet the evolution of these conditions continues. For example, new drugs for MS treatment, such as Bruton’s tyrosine kinase inhibitors (BTKi) and several candidate neuroprotective compounds, have achieved some success in phase II clinical trials (65), but their effectiveness remains to be fully evaluated. Treatment options for SLE include non-steroidal anti-inflammatory drugs, antimalarials, corticosteroids, immunosuppressants, and biologics (66). Despite the approval of new treatments, the prognosis for SLE patients remains poor (67–70). The primary goal of HT treatment is to control hypothyroidism, including the administration of synthetic levothyroxine (L-T4) (71). The etiology and pathogenic mechanisms of these three ADs are still not fully understood. Through Mendelian randomization studies, we have identified potential causal relationships between certain cytokines and these diseases at the genetic level, uncovering new targets not previously explored in immunological disease research, offering new insights for disease diagnosis and therapy.

The prevailing MR research on cytokines and autoimmune diseases typically incorporates up to 41 cytokines or focuses on a single disease outcome (72, 73). A significant strength of our study is the use of the most extensive dataset to date, comprising 91 circulating cytokines. This breadth allows for a comprehensive genetic examination of the immune networks involved in three major autoimmune diseases, enhancing our understanding of their pathogenesis. We employed bidirectional MR analysis and multiple sensitivity analyses to ensure the robustness and generalizability of our methods.

However, our study is not without limitations. The GWAS data for cytokines are relatively small, necessitating a selection threshold of *P*< 5×10^–6^ for IVs. This threshold might introduce weaker associations into the analysis, posing a risk of bias. Utilizing multiple GWAS datasets for the same phenotype could enhance result reliability, a direction for future research. Some diseases exhibit sex-related prevalence differences, such as SLE, which is more common in women (74), and MS, which has a 2–3 times higher incidence in women than in men (75). Our analysis was conducted using a limited dataset with stratified validation. Despite the considerable sample size, the exact number of positive cases remains undetermined. If we calculate based on previously mentioned prevalence rates, the resultant case count may still be relatively low, and the proportion of cases in the statistical data following gender stratification could be minimal. The inclusion of self-reported diagnoses among these cases might introduce potential biases in our results. Future objectives include replicating this research with a broader and more diverse sample to allow for more detailed stratified analysis. Furthermore, it is imperative to study the states of the diseases and the categorization of phenotypes, which would offer insights into the differential impacts of cytokines on the various dimensions and subtypes of these disorders. This analysis predominantly relies on samples of European descent, incorporating minimal data from East Asian populations. Such a demographic focus may limit the generalizability of our findings, suggesting that our conclusions might not extend fully to other ethnic or racial groups. The paucity of non-European GWAS data appropriate for our research objectives precluded an effective cross-population validation of these results. Lastly, despite employing strict Bonferroni correction for multiple testing, only some reverse MR results remained significant after adjustment. Therefore, larger GWAS datasets are needed to replicate these MR findings.

## Conclusion

Our findings suggest potential causal relationships between certain circulating cytokines and MS, SLE, and HT, including IL-18, ADA, SCF, IL-4, IL-7, IL-10RA, CXCL5, FGF23, MCP4, IL-33, CDCP1, and TRAIL. These biomarkers may serve as new targets for the diagnosis and prevention of these diseases. Additionally, our study has identified some markers not previously researched within these diseases, setting the stage for future experimental studies to further validate causal relationships, mechanisms, and therapeutic potentials.

## Data availability statement

Publicly available datasets were analyzed in this study. This data can be found here: https://www.ebi.ac.uk/gwas/.

## Author contributions

JJ: Writing – original draft, Writing – review & editing, Conceptualization, Software. YG: Writing – review & editing. SL: Conceptualization, Writing – original draft. XY: Supervision, Writing – original draft. KG: Visualization, Writing – original draft.

## Supplementary material

Supplementary Figure 1 Scatter plots depicting the genetic associations between cytokines and MS, constructed using various MR methods.

Supplementary Figure 2 Scatter plots depicting the genetic associations between cytokines and SLE, constructed using various MR methods.

Supplementary Figure 3 Scatter plots depicting the genetic associations between cytokines and HT, constructed using various MR methods.

Supplementary Figure 4 Plots of leave-one-out analyses to assess the stability of the MS results.

Supplementary Figure 5 Plots of leave-one-out analyses to assess the stability of the SLE results.

Supplementary Figure 6 Plots of leave-one-out analyses to assess the stability of the HT results.

Supplementary Figure 7 Plots of leave-one-out analyses to assess the stability of inverse MS results.

Supplementary Figure 8 Plots of leave-one-out analyses to assess the stability of inverse SLE results.

Supplementary Figure 9 Plots of leave-one-out analyses to assess the stability of inverse HT results.
