# Supplementary figures and images for: Genetically predicted associations between circulating cytokines and autoimmune diseases: a bidirectional two-sample Mendelian randomization
Source: Front Immunol. 2024 May 27;15:1404260. doi: 10.3389/fimmu.2024.1404260 (PMC11163916; doi:10.3389/fimmu.2024.1404260)

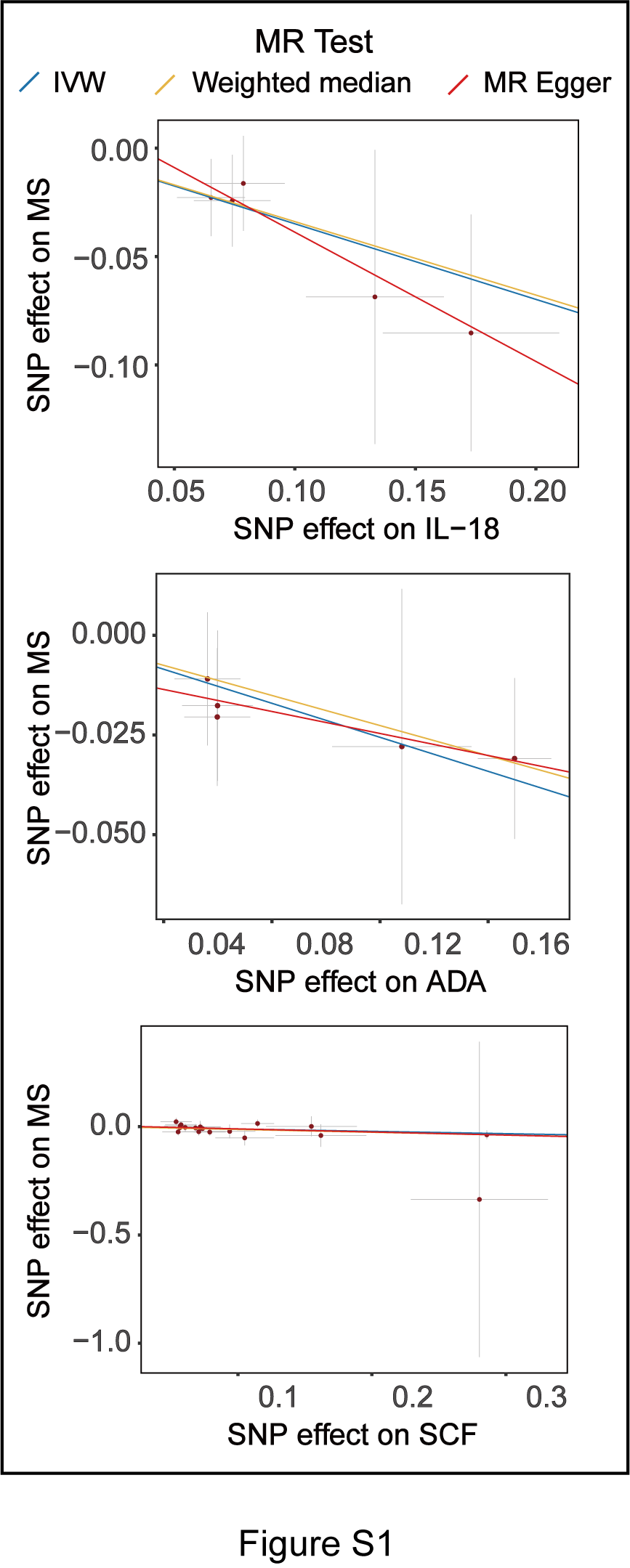

Supplement: Supplementary Figure 1 — Scatter plots depicting the genetic associations between cytokines and MS, constructed using various MR methods. [file DataSheet_1.zip › Figure S1.tif]

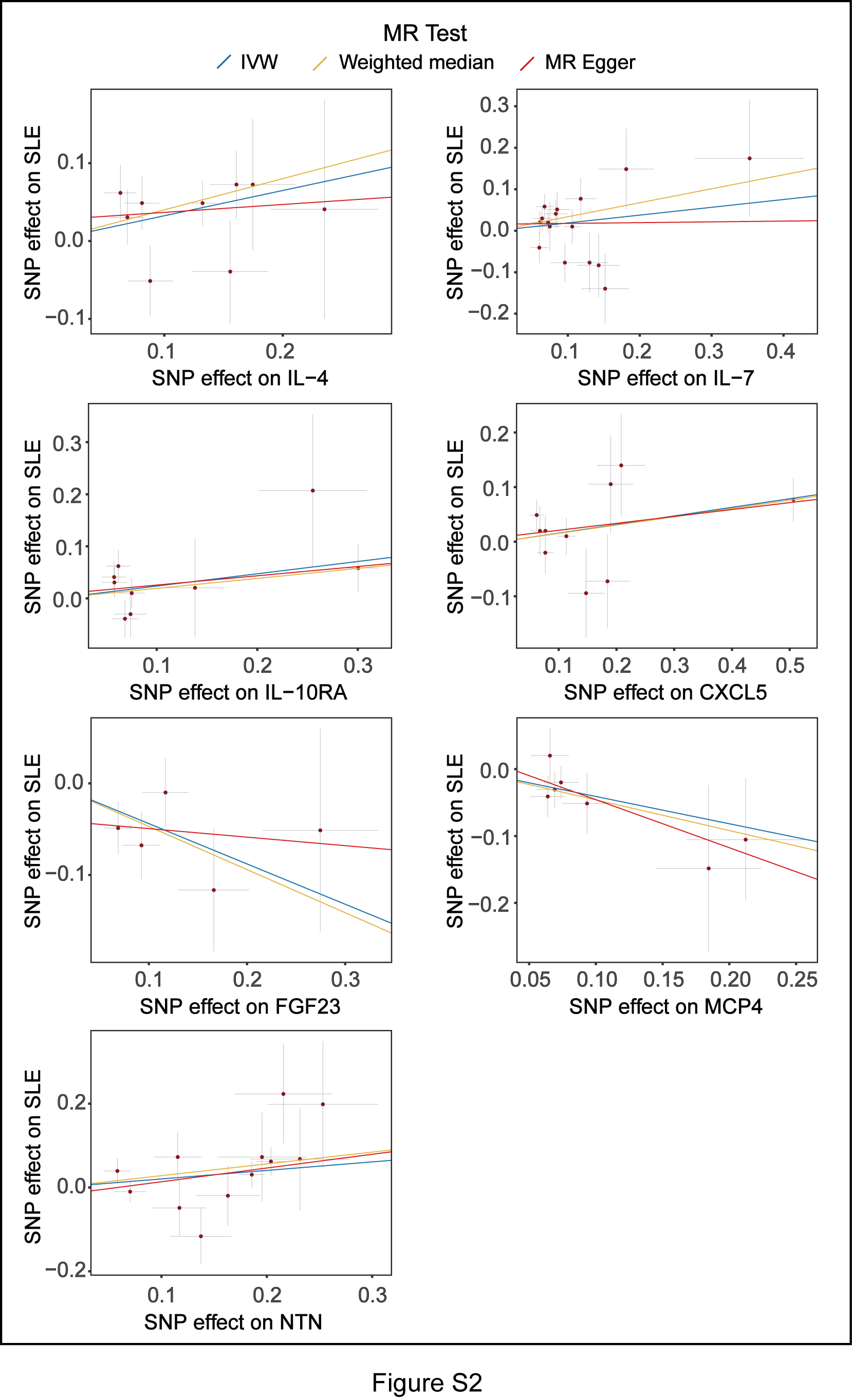

Supplement: Supplementary Figure 1 — Scatter plots depicting the genetic associations between cytokines and MS, constructed using various MR methods. [file DataSheet_1.zip › Figure S2.tif]

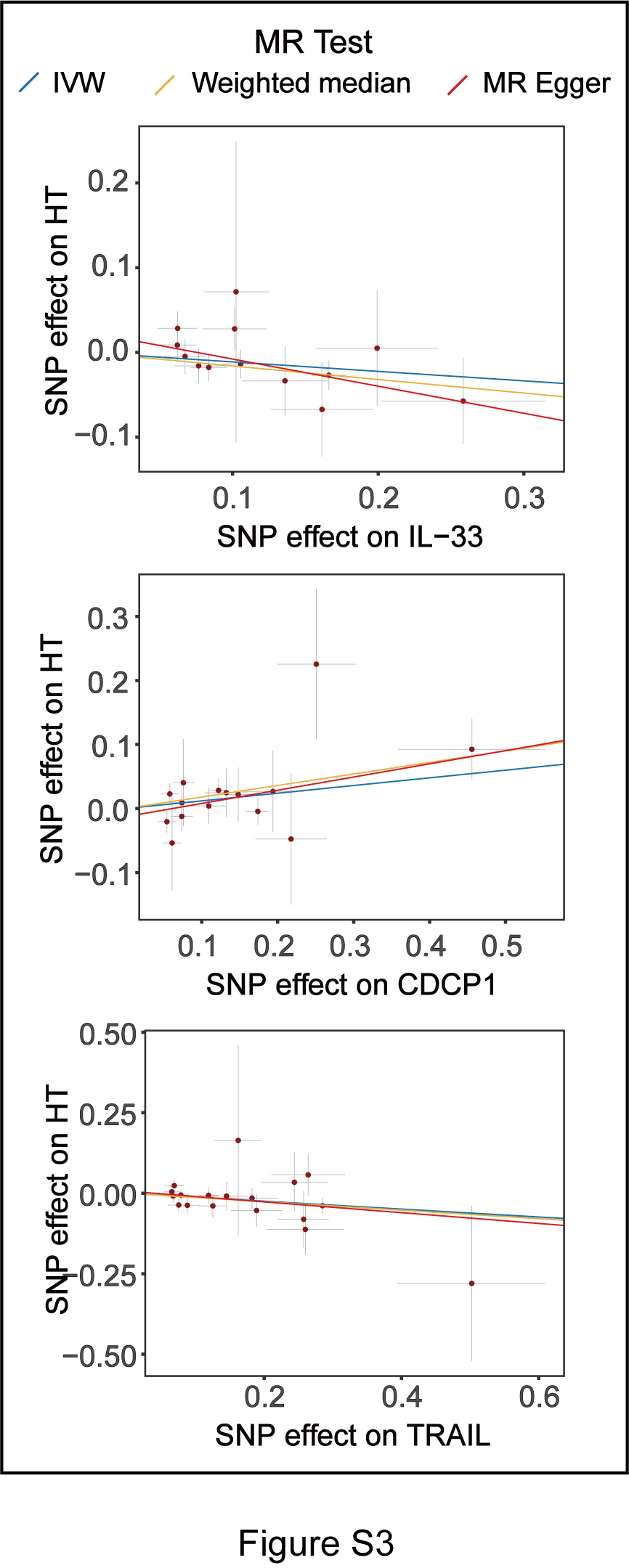

Supplement: Supplementary Figure 1 — Scatter plots depicting the genetic associations between cytokines and MS, constructed using various MR methods. [file DataSheet_1.zip › Figure S3.tif]

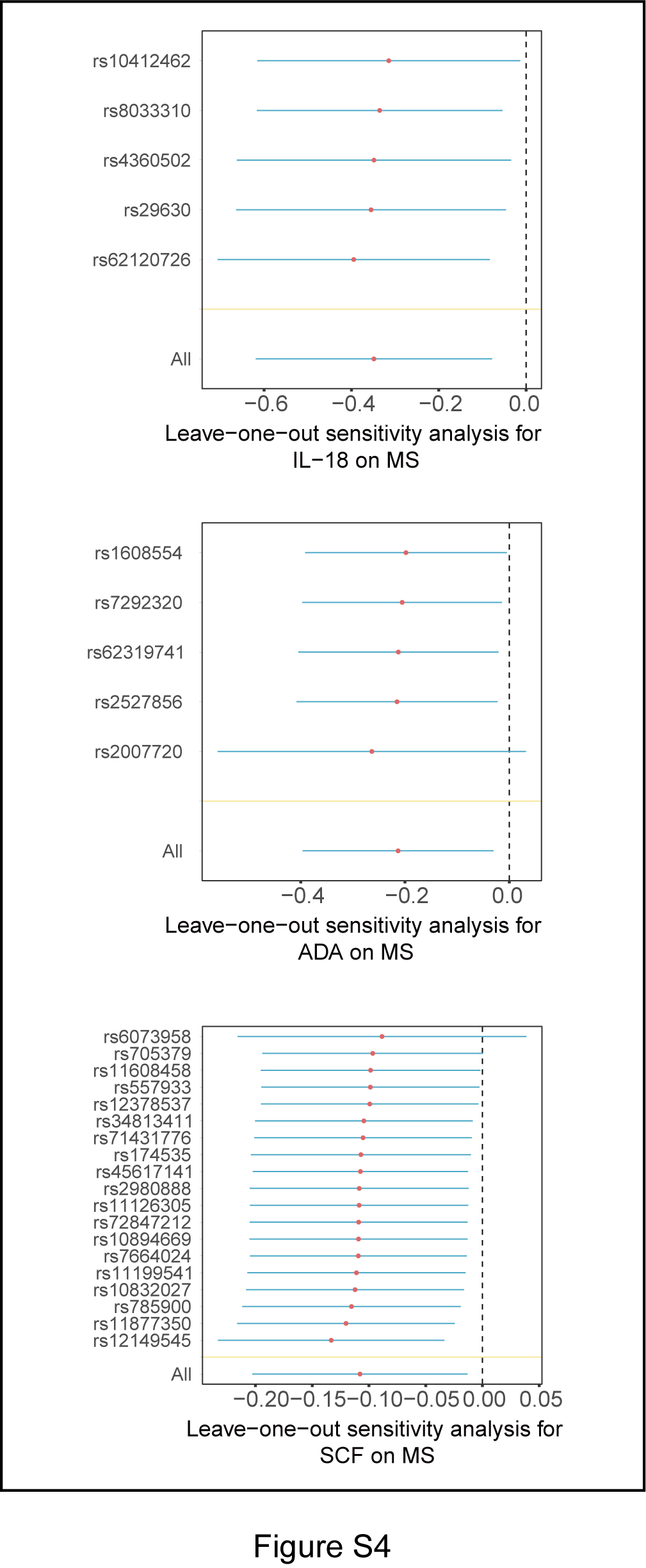

Supplement: Supplementary Figure 1 — Scatter plots depicting the genetic associations between cytokines and MS, constructed using various MR methods. [file DataSheet_1.zip › Figure S4.tif]

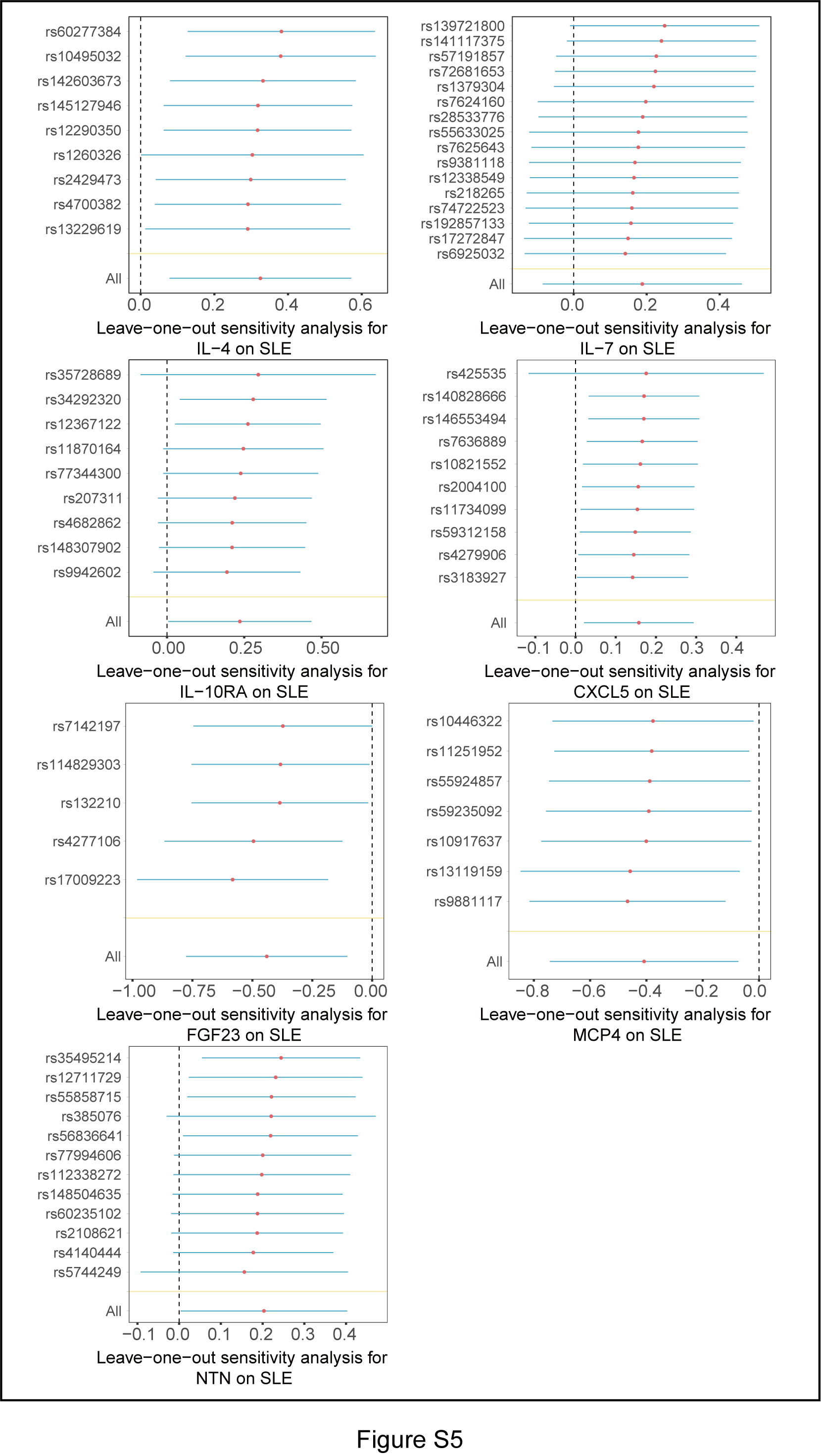

Supplement: Supplementary Figure 1 — Scatter plots depicting the genetic associations between cytokines and MS, constructed using various MR methods. [file DataSheet_1.zip › Figure S5.tif]

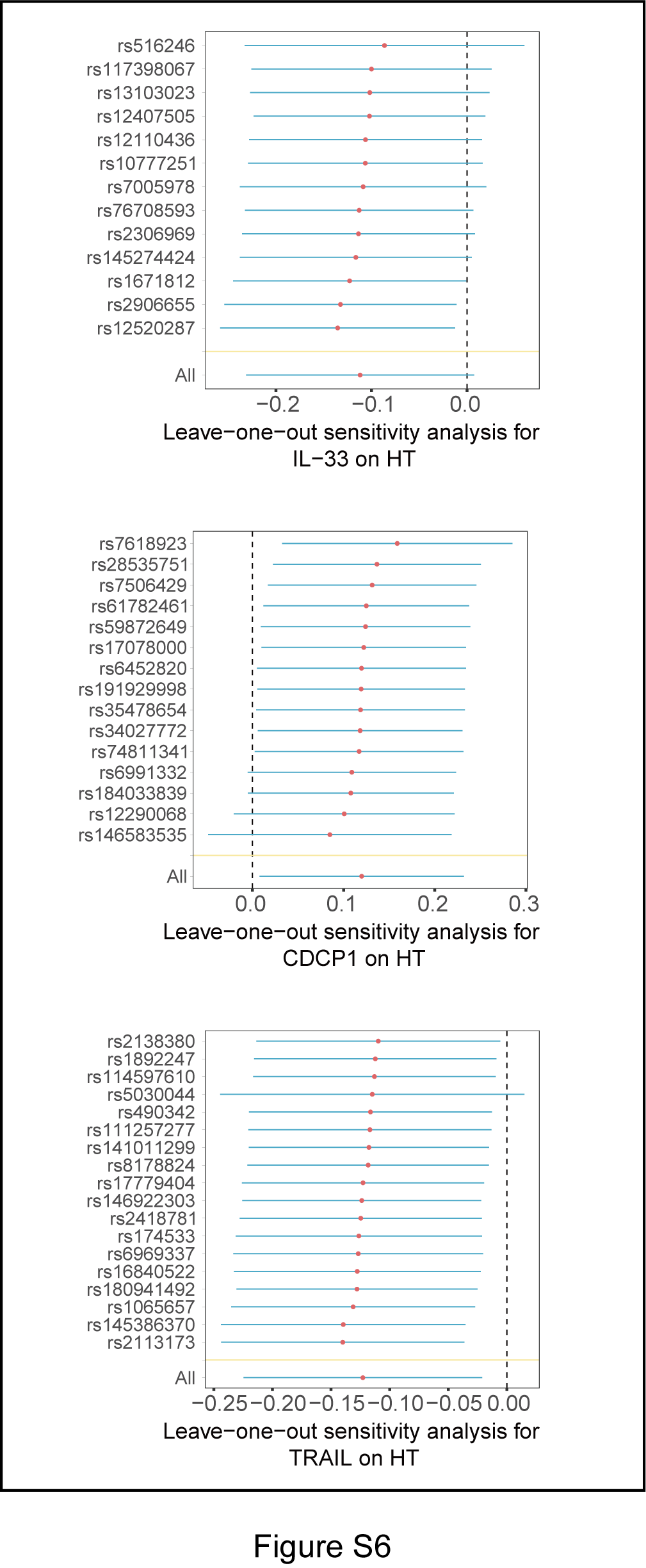

Supplement: Supplementary Figure 1 — Scatter plots depicting the genetic associations between cytokines and MS, constructed using various MR methods. [file DataSheet_1.zip › Figure S6.tif]

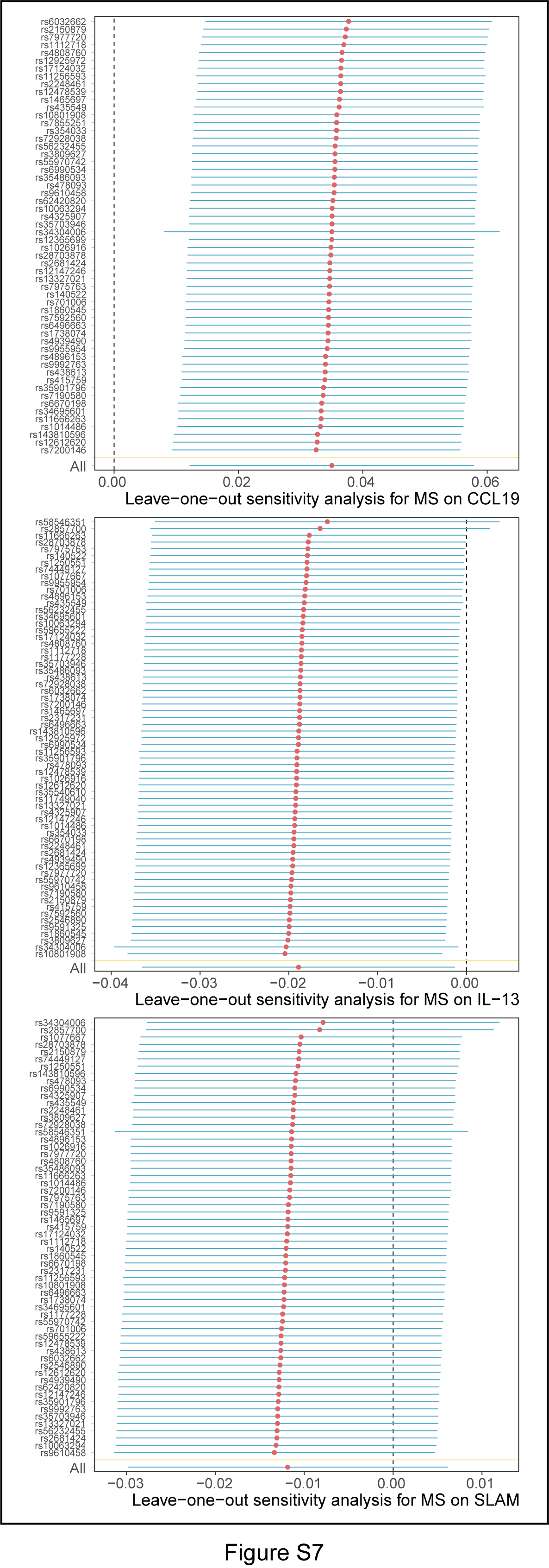

Supplement: Supplementary Figure 1 — Scatter plots depicting the genetic associations between cytokines and MS, constructed using various MR methods. [file DataSheet_1.zip › Figure S7.tif]

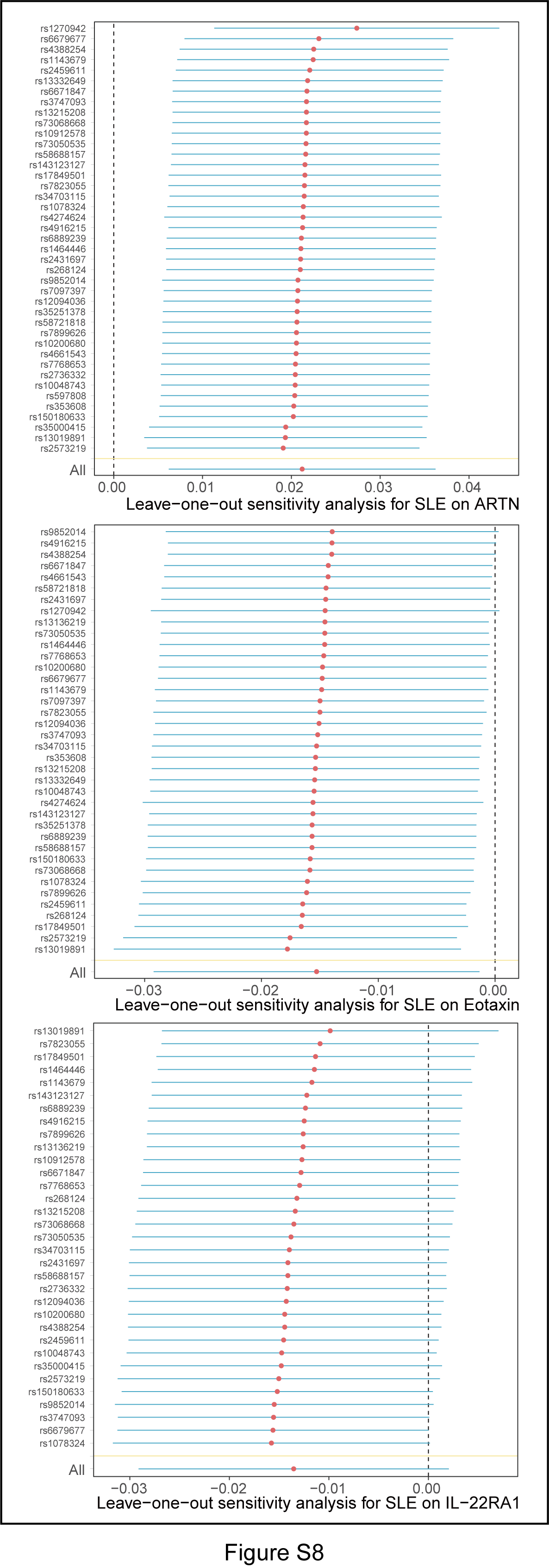

Supplement: Supplementary Figure 1 — Scatter plots depicting the genetic associations between cytokines and MS, constructed using various MR methods. [file DataSheet_1.zip › Figure S8.tif]

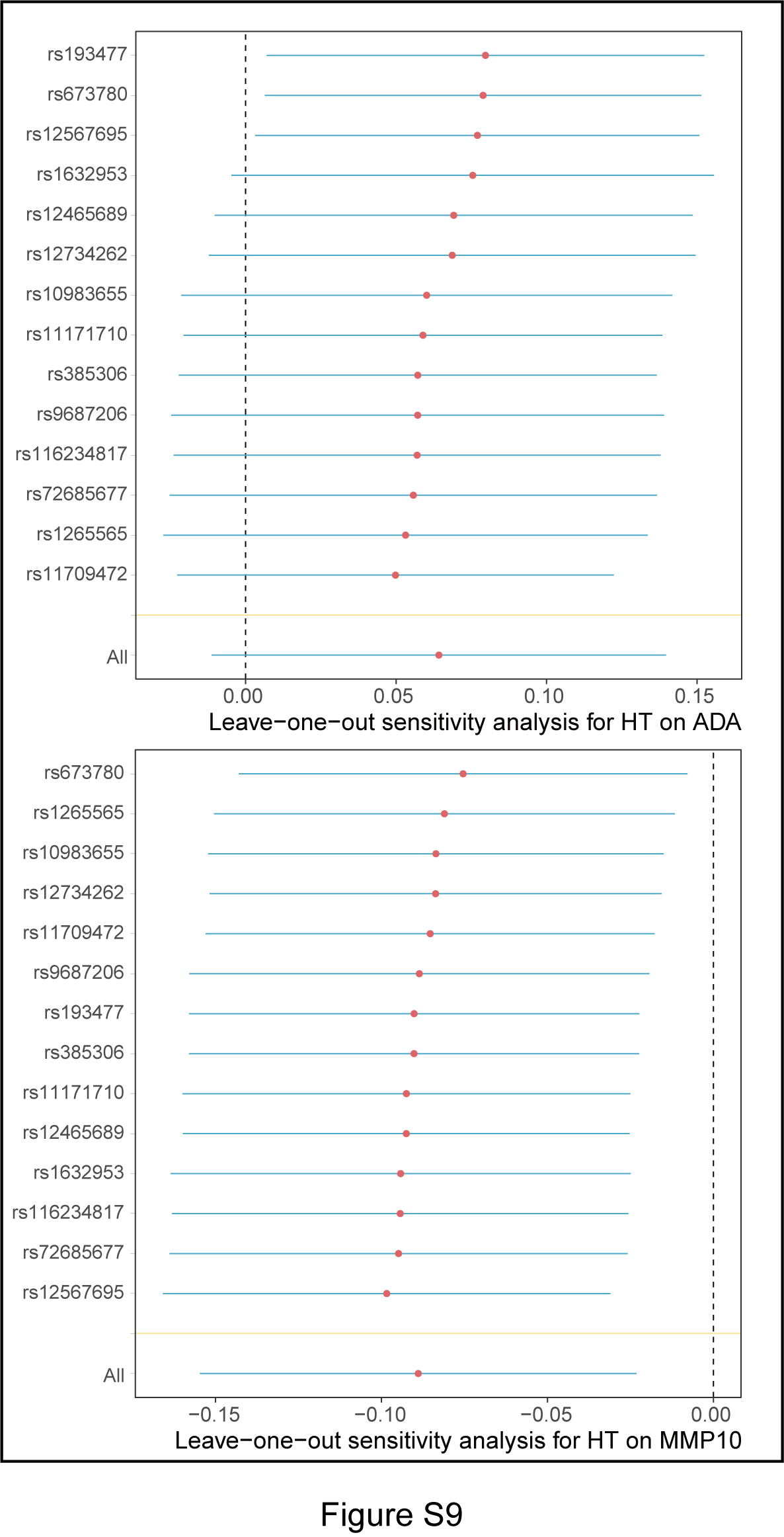

Supplement: Supplementary Figure 1 — Scatter plots depicting the genetic associations between cytokines and MS, constructed using various MR methods. [file DataSheet_1.zip › Figure S9.tif]
